# Drug Repurposing Approach against Novel Coronavirus Disease (COVID-19) through Virtual Screening Targeting SARS-CoV-2 Main Protease

**DOI:** 10.3390/biology10010002

**Published:** 2020-12-23

**Authors:** Kamrul Hasan Chowdhury, Md. Riad Chowdhury, Shafi Mahmud, Abu Montakim Tareq, Nujhat Binte Hanif, Naureen Banu, A. S. M. Ali Reza, Talha Bin Emran, Jesus Simal-Gandara

**Affiliations:** 1 Department of Pharmacy, International Islamic University Chittagong, Chittagong 4318, Bangladesh; kamrulhasan73132@gmail.com (K.H.C.); riadchy01@gmail.com (M.R.C.); montakim0.abu@gmail.com (A.M.T.); nujhataanchol@gmail.com (N.B.H.); naureen2021@gmail.com (N.B.); 2 Microbiology Laboratory, Bioinformatics Division, Department of Genetic Engineering and Biotechnology, University of Rajshahi, Rajshahi 6205, Bangladesh; shafimahmudfz@gmail.com; 3 Department of Pharmacy, BGC Trust University Bangladesh, Chittagong 4381, Bangladesh; 4 Nutrition and Bromatology Group, Department of Analytical and Food Chemistry, Faculty of Food Science and Technology, University of Vigo–Ourense Campus, E32004 Ourense, Spain

**Keywords:** COVID-19, antiviral agents, drug repurposing, molecular docking, molecular dynamics, E-pharmacophore hypothesis, virtual screening

## Abstract

**Simple Summary:**

With the urgent necessity of potential treatment against novel coronavirus disease, we used several computational methods to search for active drugs from an extensive database. The results of our investigation suggested several established drugs that can be subjected to further analysis for the treatment of novel coronavirus disease. Various methods used in this study proved the effectiveness of the retrieved drugs. Therefore, our findings highly recommend the mentioned drugs to be scrutinized to discover drugs against novel coronavirus.

**Abstract:**

Novel coronavirus disease (COVID-19) was identified from China in December 2019 and spread rapidly through human-to-human transmission, affecting so many people worldwide. Until now, there has been no specific treatment against the disease and repurposing of the drug. Our investigation aimed to screen potential inhibitors against coronavirus for the repurposing of drugs. Our study analyzed sequence comparison among SARS-CoV, SARS-CoV-2, and MERS-CoV to determine the identity matrix using discovery studio. SARS-CoV-2 M^pro^ was targeted to generate an E-pharmacophore hypothesis to screen drugs from the DrugBank database having similar features. Promising drugs were used for docking-based virtual screening at several precisions. Best hits from virtual screening were subjected to MM/GBSA analysis to evaluate binding free energy, followed by the analysis of binding interactions. Furthermore, the molecular dynamics simulation approaches were carried out to assess the docked complex’s conformational stability. A total of 33 drug classes were found from virtual screening based on their docking scores. Among them, seven potential drugs with several anticancer, antibiotic, and immunometabolic categories were screened and showed promising MM/GBSA scores. During interaction analysis, these drugs exhibited different types of hydrogen and hydrophobic interactions with amino acid residue. Besides, 17 experimental drugs selected from virtual screening might be crucial for drug discovery against COVID-19. The RMSD, RMSF, SASA, Rg, and MM/PBSA descriptors from molecular dynamics simulation confirmed the complex’s firm nature. Seven promising drugs for repurposing against SARS-CoV-2 main protease (M^pro^), namely sapanisertib, ornidazole, napabucasin, lenalidomide, daniquidone, indoximod, and salicylamide, could be vital for the treatment of COVID-19. However, extensive in vivo and in vitro studies are required to evaluate the mentioned drug’s activity.

## 1. Introduction

Novel coronavirus disease (COVID-19) has become a threatening illness globally and was declared as a pandemic by WHO (World Health Organization). COVID-19 started spreading from the Wuhan seafood market in China since December 2019 [1]. As of the writing of this paper, 1,151,622 persons have died so far in COVID-19 and confirmed cases of persons with COVID-19 are 42,662,304 [2].

Coronavirus (CoVs) are the family of Coronaviridae, which is single-stranded enveloped positive RNA virus subdivided into alpha (α), beta (β), gamma (γ), and delta (δ). Among them, the coronavirus β (β-CoV) group is divided into severe acute respiratory syndrome coronavirus (SARS-CoV), severe acute respiratory syndrome coronavirus-2 (SARS-CoV-2), and middle east respiratory syndrome coronavirus (MERS-CoV) [3]. These three types of virus are highly fatal and responsible for respiratory, liver, gastrointestinal, and central nervous system damage in humans and animals [4]. In case of respiratory disease, it can lead to severe pneumonia with several symptoms, including fever, dry cough, vomiting, fatigue, diarrhea, and shortness of breath [5,6].

SARS-CoV-2 responsible for COVID-19 is extremely infectious and more pathogenic than SARS-CoV and MERS-CoV, which can transmit from human to human and cause fatal illness [5]. As there are no potential drugs or vaccines against coronavirus until now, emergency investigations are required to establish effective treatment. Several groups of drugs are being investigated against COVID-19, which includes drugs, namely hydroxychloroquine, chloroquine, lopinavir, ritonavir, remdesivir, etc., used for the treatment of SARS-CoV, MERS-CoV, and other viruses. However, there is an emergency requirement to establish novel, selective, and potential inhibitors against COVID-19 for the effective treatment [7]. In this short period of time, repurposing of drugs is a significant way to find out potential inhibitors against COVID-19. During repurposing, virtual screening, pharmacophore modeling, and other computational methods are extensively used [8].

The main protease (M^pro^/3CL^pro^) is an attractive drug target due to its vital function that regulates polyproteins translated from the viral RNA [9]. The aim of our study was to focus on the SARS-CoV-2 main protease as a potential drug target to screen drugs for repurposing against COVID-19. The viral protein with a bound inhibitor was subjected to E-pharmacophore and molecular docking-based virtual screening to determine promising inhibitors of novel coronavirus. In addition, potential drugs were investigated through MM/GBSA and binding interaction for further analysis.

## 2. Materials and Methods

### 2.1. Sequence Comparison

The crystal structures of SARS-CoV-2 M^pro^ (PDB ID: 5R7Y) [10], SARS-CoV M^pro^ (PDB ID: 2C3S) [11], and MERS-CoV M^pro^ (PDB ID: 5C3N) [12] were obtained from protein data bank. Afterwards, the protein structures were transferred into discovery studio software in order to align and explore their structural sequence for comparison purposes [13]. Accordingly, SARS-CoV-2 main protease (PDB ID: 5R7Y) in complex with Z45617795 at a resolution of 1.65 Å was used for further investigation.

### 2.2. Protein Preparation

Pre-processing of the structure of the COVID-19 main protease in complex with Z45617795 was carried out through transformation of selenomethionines into methionines, removal of water molecules, and addition of missing hydrogen atoms followed by minimization of the structure utilizing the OPLS3 force field [14]. All these steps were performed utilizing the protein preparation wizard of Schrödinger Maestro (Protein Preparation Wizard; Epik, Schrödinger, LLC, New York, NY, USA; v.11.1) [15]. In a similar manner, COVID-19 main protease in complex with inhibitor N3 at 1.7 angstrom (PDB ID: 7 BQY) was prepared.

### 2.3. Generation of E-Pharmacophore Hypothesis

Previously prepared protein was subjected to E-pharmacophore hypothesis establishment using phase module of Schrodinger suit (Phase, Schrödinger, LLC, New York, NY, USA; v.11.1) to generate the pharmacophoric features [16]. Different features, namely aromatic ring (R), hydrogen bond acceptor (A), hydrogen bond donor (D), positive ion (P), negative ion (N), and hydrophobicity (H), were mapped to form the protein-ligand complex.

### 2.4. Virtual Screening using E-Pharmacophore Hypothesis

The molecular structure of 8820 drugs from the DrugBank database were screened for promising candidates against SARS-CoV-2 main protease (M^pro^) enzyme utilizing the pharmacophoric features from the established E-pharmacophore hypothesis [17]. E-pharmacophore-based screening was carried out by the phase ligand screening module of Schrodinger Suit [16].

### 2.5. Ligand Preparation

Best hits obtained from virtual screening were prepared utilizing LigPrep module of Schrodinger suit [18]. Selected drugs were subjected to an optimization process at target pH (7 ± 1) in order to generate all possible states, such as tautomeric and stereo-isomeric followed by minimization at the OPLS3 force field [14].

### 2.6. Virtual Screening Based on Molecular Docking

A glide grid box was generated at the receptor complex site at 15 × 15 × 15 Å along the X, Y, and Z axes while docking. Virtual screening using molecular docking was performed by virtual screening workflow of Schrodinger suit to carry out high-throughput virtual screening (HTVS), standard precision (SP), and extra precision (XP) docking, respectively. Docking of each precision was filtrated and expressed as a glide score [19]. Subsequently, the ligand (Z45617795) bound to SARS-CoV-2 main protease was extracted from protein-ligand complex and subjected to re-docking through standard precision (SP) docking [20]. From this, the docking score was obtained as a control (control 1) to compare the value with newly screened drugs. Additionally, inhibitor N3 was docked with the COVID-19 M^pro^ in complex with inhibitor N3 (control 2).

### 2.7. MM/GBSA and Interaction Analysis

Promising candidates obtained from docking-based virtual screening were subjected to MM/GBSA using Prime MM/GBSA module of Schrodinger Suit to estimate binding free energy and compared with control 1 (Z45617795) and control 2 (inhibitor N3) [21]. Best candidates from MM/GBSA analysis were used for further interaction analysis by utilizing discovery studio software [13].

### 2.8. Molecular Dynamics Simulations

The molecular dynamics simulation of the main protease of SARS-CoV-2 and screened ligand complexes were further analyzed through molecular dynamics simulation to evaluate their motion and structural integrity in the YASARA software package [22]. Here, the compound Z and the main protease complex (PDB ID: 5R7Y), and inhibitor N3 along with main protease protein (PDB ID: 7BQY) were considered as control 1 and control 2. The drug-protein complexes were initially cleaned in the software and the hydrogen bond network of the system was optimized. The AMBER14 force field [23] was applied and a cubic simulation cell was created. The cell size was set bigger than the drug-protein complex by 20 Å in all cases so that each biological system can move freely. The initial energy minimization procedure was applied through a simulated annealing method by employing the steepest gradient approaches. The simulation cell box was neutralized with the addition of water molecules and 0.9% NaCl at 7.4 pH. The temperature of each system was set as 298k and the Berendsen thermostat was used to control the cell temperature [24]. The long-range electrostatic interactions of the system were calculated through PME or the Particle Mesh Ewald method at a cut off radius 8 Å. The time-step of the simulation cell was set as 1.25 fs and each of the trajectories were saved at a 100-ps interval. Finally, the simulation was carried out for 100 ns to analyze the root mean square deviation (RMSD), root mean square fluctuation (RMSF), radius of gyration (Rg), solvent accessible surface area (SASA), and hydrogen bond [25,26,27,28].

The MM/PBSA method was applied to calculate the binding free energy from simulation trajectories. The default macro of YASARA program (md_analyzebindenergy.mcr) was modified and free energy calculations was performed for the seven drug-protein complexes. The following equation was used to calculate MM/PBSA binding free energy:

ΔGbind = ΔGcomplex (minimized) − [ΔGligand (minimized) + ΔGreceptor (minimized)](1)

ΔGbind = ΔG MM +ΔG PB +ΔG SA − TΔS(2)

where ΔG MM is the sum of Van der Waals and electrostatic interaction, ΔG PB and ΔG SA is the polar and non-polar solvation energies, and TΔS is the entropic contribution.

## 3. Results and Discussion

### 3.1. Sequence Comparison

**Figure 1:**
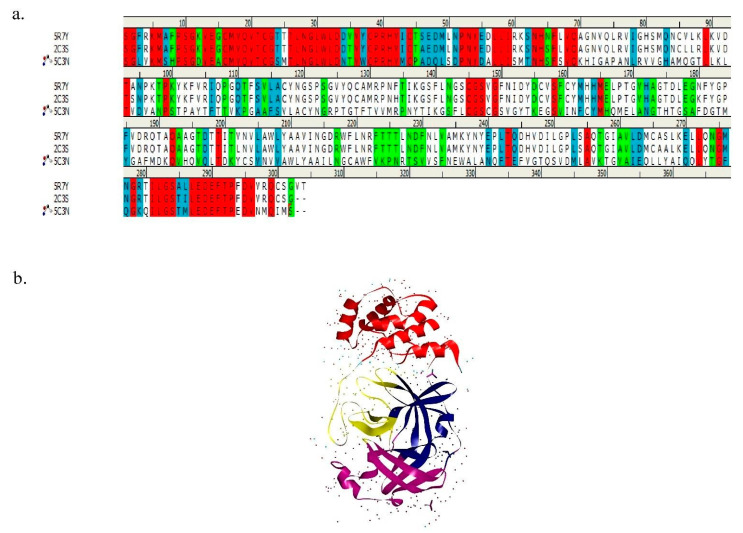
Sequence comparison matrix of COVID-19 proteins. (**a**) Sequence comparison between SARS-CoV-2 (PDB ID: 5R7Y), SARS-CoV (PDB ID: 2C3S), and MERS-CoV (PDB ID: 5C3N). (**b**) Primary structure of SARS-CoV-2 M^pro^ (PDB ID: 5R7Y).

Comparison of SARS-CoV-2 M^pro^ (PDB ID: 5R7Y), SARS-CoV M^pro^ (PDB ID: 2C3S), and MERS-CoV M^pro^ (PDB ID: 5C3N) sequences are represented in Figure 1a. The red color denotes identically matching, light blue refers to as strongly matching, green color indicates weakly matching, and sequence without any color signifies as non-matching with each other. The identity matrix (%) of the sequence comparison is demonstrated in Table 1. In the comparison with SARS-CoV-2, 96% and 24.2% identity matrix were detected for SARS-CoV and MERS-CoV, respectively, and SARS-CoV-2 M^pro^ suggested an attractive drug target for the treatment of COVID-19 due to its similarity with SARS-CoV and MERS-CoV. The identity matrix indicates an effective target for the screening of promising drugs to inhibit coronavirus replication into the host cell. Previous studies suggest that the amino acid difference between SARS-CoV-2 and SARS-CoV was 12. On the other hand, amino acid residue conservation between SARS-CoV-2 and MERS-CoV was 153 [8]. Structural analysis of the SARS-CoV-2 M^pro^ active site revealed several amino acid residues, namely THR-25, THR-190, THR-26, HIS-41, HIS-163, LEU-27, LEU-141, LEU-166, SER-46, PHE-140, PHE-185, PRO-168, MET-49, MET-165, TYR-54, ASN-142, ALA-191, GLY-143, GLN-189, GLN-192, GLU-166, ASP-187, and CYS-145 [29]. The primary structure of SARS-CoV-2 M^pro^ is presented in Figure 1b.

### 3.2. The E-Pharmacophore Hypothesis

**Figure 2:**
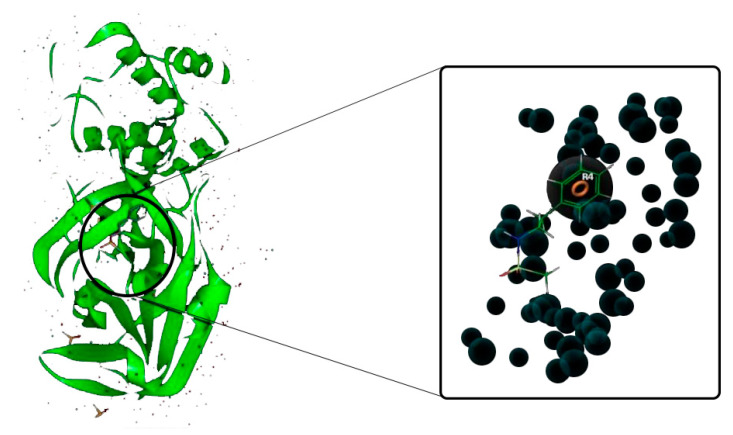
The E-pharmacophore feature (R4) of SARS-CoV-2 (PDB ID: 5R7Y) M^pro^ complex with Z45617795.

A pharmacophore is a molecular feature required to detect a ligand molecularly. Pharmacophore models can also be used to identify new ligands that bind to the same receiver through virtual screening [30]. In the current study, the pharmacophore hypothesis was established using the main protease of SARS-CoV-2 (PDB ID: 5R7Y) in complex with Z45617795. The interactions of complex can lead to the generation of pharmacophore features, which can be targeted for the screening of candidates with similar features. The E-pharmacophore hypothesis displayed only one feature, which was one aromatic ring (R4). The aromatic ring (R4) at the complex site is exhibited in Figure 2.

### 3.3. Virtual Screening Using E-Pharmacophore Hypothesis

About 1000 drugs from DrugBank database were screened based on the E-pharmacophore hypothesis, where the candidates were grouped as approved, investigational, and experimental drugs (data not shown). Best hits in terms of high relatedness with the E-pharmacophore hypothesis were obtained from the entire database.

### 3.4. Virtual Screening Based on Molecular Docking

To provide a more valid result of virtual screening, a protein structure is required with high resolution [30]. The protein structure analyzed in this study has a resolution of 1.65 Å and was used for further docking-based virtual screening. Using HTVS, SP, and XP docking, 1000 drugs obtained from pharmacophore-based virtual screening were rescreened through filtering and removing low-scoring drugs at each step. Approved and investigational drugs were segregated from the HTVS, SP, and XP pool based on the docking score and categorized as analgesic, antibiotic, anticonvulsants, benzene derivatives, anticoagulant, vitiligo, pyrimidines, anticancer, antiviral, cardiovascular agent, and immunometabolic drugs (Table 2). All candidates showed higher docking scores in comparison with control 1 (−5.367 kcal/mol). This may conclude that the following 16 approved and investigational drugs are the most promising in terms of molecular docking. To get better outcomes, subsequently, we performed the MM/GBSA and molecular dynamics simulation for further clarification.

In addition, 17 experimental drugs higher than the reference docking score (−5.370 kcal/mol) were separated from the results of virtual screening (Table 3). Among these drugs, DB02502, DB02309, and DB02690 showed the best docking score. Though these drugs are not approved or evaluated for the treatment purpose, the candidates are still valuable for noble drug discovery pipeline against COVID-19 through in vitro, in vivo, and other mechanistic investigations.

### 3.5. MM/GBSA and Interaction Analysis

**Figure 3:**
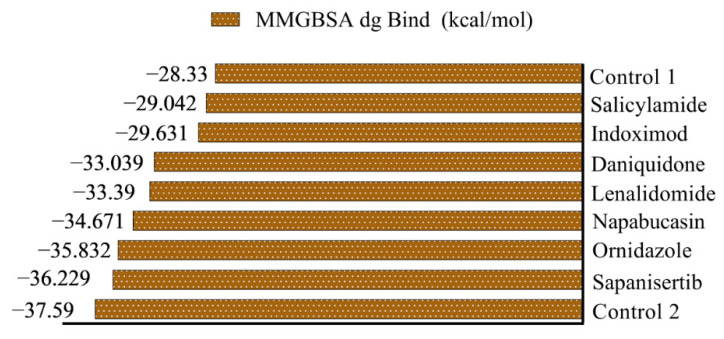
Best drugs from MM/GBSA analysis with MM/GBSA dg Bind score.

A total of 16 approved and investigational drugs were subjected to MM/GBSA analysis to evaluate binding free energy and for comparison with the control (Z45617795). There was a good correlation between MMGBSA dg Bind and binding affinity, where a more negative value suggests stronger binding affinity [31]. From the result of MM/GBSA analysis, seven drugs were found to possess a higher MM/GBSA score than the control Z45617795 (−28.33 kcal/mol) (Figure 3). The drugs with promising MM/GBSA dg bind scores are sapanisetrib (−36.229 kcal/mol), ornidazole (−35.832 kcal/mol), napabucasin (−34.671 kcal/mol), lenalidomide (−33.390 kcal/mol), daniquidone (−33.039 kcal/mol), indoximod (−29.631 kcal/mol), and salicylamide (−29.042 kcal/mol). The binding interactions of these candidates are exhibited in Table 4.

**Figure 4:**
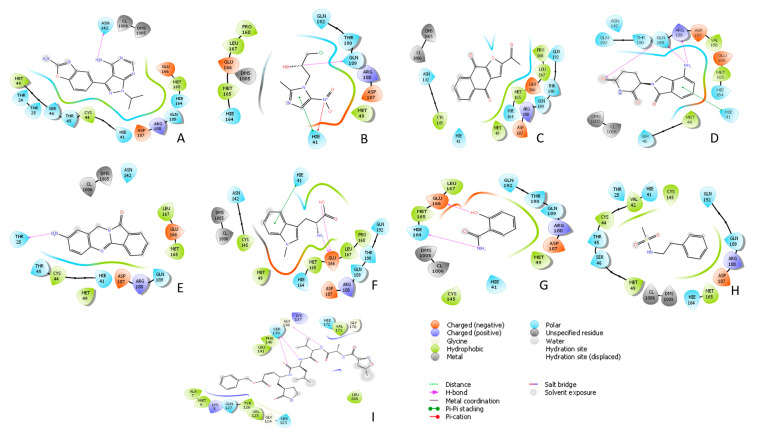
2D docking interactions of (**A**) Sapanisertib, (**B**) Ornidazole, (**C**) Napabucasin, (**D**) Lenalidomide, (**E**) Daniquidone, (**F**) Indoximod, (**G**) Salicylamide, and (**H**) Control 1 with SARS-CoV-2 M^pro^ (PDB ID: 5R7Y); (**I**) Control 2 with SARS-CoV-2 M^pro^ (PDB ID: 7BQY). The colors indicate the residue type: red-acidic, green-hydrophobic, purple-basic, blue-polar, light gray-other, darker gray-metal atoms. Interactions with the protein are marked with lines between ligand atoms and protein residues: solid pink—H-bonds to the protein backbone, dotted pink-H-bonds to protein side chains, green—pi-pi stacking interactions, orange-pi-cation interactions. Ligand atoms that are exposed to solvent are marked with gray spheres. The protein “pocket” is displayed with a line around the ligand, colored with the color of the nearest protein residue. The gap in the line shows the opening of the pocket.

Sapanisertib and napabucasin are investigational anticancer drugs, which induce apoptosis of the cancer cell [32,33]. Sapanisertib demonstrated hydrogen bond interaction with ASN-142 and hydrophobic bond interactions with HIS-41, MET-49, and MET-165 (Figure 4A). On the other hand, napabucasin formed hydrogen bond interaction with Pro-168 and hydrophobic bond interaction with MET-165 (Figure 4C). In the docking-based virtual screening, sapanisertib and napabucasin provided a −6.24, and −6.25 kcal/mol docking score, respectively, against SARS-CoV-2. Daniquidone is another investigational anticancer drug that inhibits DNA replication along with the inhibition of RNA and protein synthesis [34]. Daniquidone inhibits SARS-CoV-2 with a −6.13 kcal/mol docking score that exhibited a key hydrogen bond interaction with THR-25 and hydrophobic bond interactions with HIS-41, MET-49, and MET-165 (Figure 4E). Lenalidomide acts by destroying tumor cells and possesses an immunomodulatory effect [35]. It is an approved anticancer drug with a −5.99 kcal/mol docking score and suggested LYS-5, ARG-4 amino acid residue interactions through the hydrogen bond and ALA-7 amino acid residue interaction through the hydrophobic bond (Figure 4D). Additionally, ornidazole is used against infectious disease, which interrupts the DNA replication and transcription process. It is an investigational drug that shows antibiotic activity [36]. According to this study, ornidazole showed the second best hit in MM/GBSA analysis (−35.832 kcal/mol) with the second best docking score (−6.67 kcal/mol). Ornidazole was found to inhibit SARS-CoV-2 M^pro^ through hydrogen bonds, including ARG-188 and GLN-189, and hydrophobic bonds, including HIS-41 and MET-165 (Figure 4B). In addition, an investigational metabolic agent named indoximod acts by boosting immunity against infectious disease and improving response to many anticancer agents [37]. In our study, indoximod inhibited viral receptor (SARS-CoV-2) with a docking score of −6.05 kcal/mol through thehydrogen bond with THR-129 and GlU-166, and hydrophobic bond with MET-165 (Figure 4F). Lastly, salicylamide is an approved analgesic drug, which demonstrated the best docking score (−7.10 kcal/mol) amongst all approved and investigational drugs in the molecular docking-based virtual screening. Previous study suggests that salicylamide inhibits cytochromes P-450 (CYP) enzyme activity [38]. In our investigation, interaction analysis of salicylamide showed hydrogen bond interactions with CYS-145, HIS-164, and GLU-166, and hydrophobic bond interaction with MET-165 (Figure 4G). These drugs might be repurposed as an effective inhibitor of COVID-19 to stop the spreading of coronavirus replication into the host cell.

### 3.6. Molecular Dynamics Simulations

**Figure 5:**
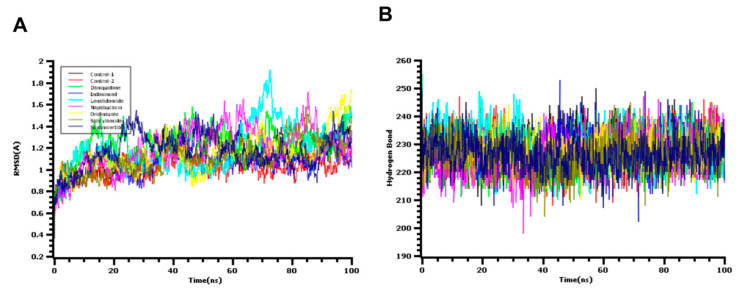
The molecular dynamics simulation study of the docked complex, (**A**) root mean square deviation (RMSD), and (**B**) hydrogen bond were assessed from the simulation trajectories.

The simulation at the atomistic scale may enable insights about macromolecules and their conformational variations to be obtained [31]. The reliability of the docking score and their validation largely depend on simulation data as the protein motion can be assessed against the function of time in the simulation box [32]. The root mean square deviation of the c-alpha atoms from M^pro^ of SARS-CoV-2 and screened drug molecules were evaluated through simulation trajectories to understand the conformational variations of the biological system against time functions. The average RMSD profile found from control 1, control 2, daniquidone, indoximod, lenalidomide, napabucasin, ornidazole, salicylamide, and sapanisertib complexes was 1.13, 1.09, 1.22, 1.08, 1.19, 1.194, 1.14, 1.11, and 1.17 Å, respectively. The lenalidomide and napabucasin were initially stable and maintains the structural integrity during most of the simulation time; however, a high rise of the RMSD value was observed for 60–80 ns time. The rest of the simulation trajectories of both complexes demonstrate a lower RMSD profile for them; however, the fluctuation level of RMSD was not too high for them. Moreover, the rest of the complexes exhibited lower RMSD values from 0.8–1.4 Å, which may be responsible for the lower level of flexibility across the simulation time. The root mean square deviation of the docked complex were found as similar with both complexes (Figure 5A). Although the pharmacophore revealed one feature, the complex on molecular dynamic simulation showed initial fluctuations in the RMSD plot for a certain period and attained equilibrium, which remained stable during the entire simulation for 100 ns [10].

On the other hand, the solvent-accessible surface area or SASA descriptors correlate with the protein volume, where a lower SASA profile specifies the condensed nature of the protein. The salicylamide and main protease complex were in a stable state from 0–20 ns, but a higher rise was observed till the entire simulation time than other complexes. This upper SASA profile may determine the expansion of the protein surface area. Comparatively, the indoximod exhibited shrinkage of the surface area from 10–20 ns and thereafter, had a similar profile like other protein-drug complexes. Although, the sapanisertib complex resembled SASA descriptors with salicylamide at the preliminary simulation phase, they did not fluctuate for the rest of the time (Figure S1A).

The degree of the protein flexibility and mobile nature of the biological systems depends on the radius of gyration values. The salicylamide and sapanisertib complex had higher Rg values like SASA descriptors. The higher level of the Rg value from both complexes may define the additional labile nature along with the loose packaging system during the simulation. This nature of both protein complexes associates with the folding and unfolding mechanism of the protein complex [39,40]. Therefore, other complexes had a comparatively lower Rg profile and revealed a more rigid nature as no significant level of deviations was found (Figure S1B). The hydrogen bond plays a vital role to provide a solid base to the biological macromolecules and systems. Therefore, the simulation trajectories were also utilized for the hydrogen bond calculation of seven drug-protein complexes. The daniquidone, indoximod, and lenalidomide complexes form more hydrogen bonds than other complexes. Although these above-mentioned three complexes had a higher hydrogen bond number, other complexes also did not show any deviations (Figure 5B). The control 1 complexes exhibited lower SASA values than all the complex, which indicates the contracted nature of this complex during simulation.

**Figure 6:**
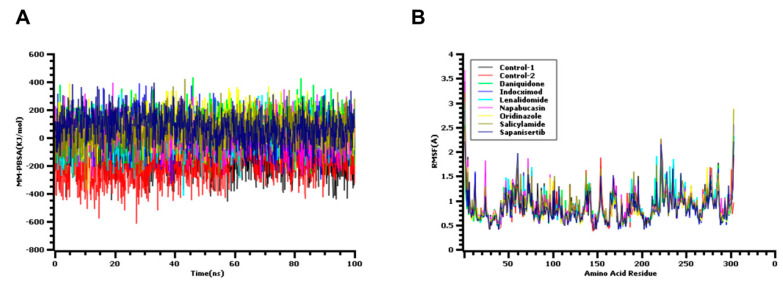
The molecular dynamics simulation study of the docked complex, (**A**) The binding free energy from MM/PBSA method, and (**B**) root mean square fluctuations (RMSF) of the amino acid residues to understand fluctuations.

The MM/PBSA method is considered stronger and more superior model for calculating binding free energy than MM/GBSA [33]. The MM/PBSA and MM/GBSA models method applies molecular mechanics and continuum solvation models. Here, both tools combinedly predict the binding free energy more efficiently in the drug designing and virtual screening process. We followed both the MM/GBSA and MM/PBSA method to have more accuracy. To concrete the docking study, the binding free energy was calculated by utilizing the MM/PBSA method. All 1000 snapshots from simulation trajectories were used for the calculations and are depicted in Figure 6A. The average binding free energy from control 1, control 2, daniquidone, indoximod, lenalidomide, napabucasin, oridinazole, salicylamide, and sapanisertib was found as 152.246, −187.096, 70.21, 23.88, 10.96, 17.81, 84.62, 33.25, and 72.55 KJ/mol, respectively, where salicylamide had the higher energy. This higher level of free energy correlates with more favorable binding with the protein. On the other hand, the degree of the protein flexibility in the amino acid sequence can be understood through the RMSF descriptors. From Figure 6B, it can be observed that every amino acid except some sequence from the main protease of SARS-CoV-2 had an RMSF value less than 2.5 Å, which reveals the lower flexibility level of every drug complex. Although all the complex exhibited a stable profile in molecular dynamics simulation, daniquidone, oridinazole, and sapanisertib showed a better performance as they had higher hydrogen bond, lower RMSD, and higher binding free energy in the simulation trajectory.

## 4. Conclusions

Pharmacophore modeling, molecular docking, virtual screening, and other computational methods are widely used to repurpose known drugs. In this investigation, sequence comparison of SARS-CoV-2 M^pro^ showed a high identity matrix with SARS-CoV and slide identity matrix with MERS-CoV. Further virtual screening based on molecular docking revealed 33 different drug groups of several categories. Among this, 16 approved and investigational drugs were found to inhibit SARS-CoV-2. In addition, MM/GBSA analysis reported seven drugs, namely sapanisertib, ornidazole, napabucasin, lenalidomide, daniquidone, indoximod, and salicylamide, that showed higher dg Bind scores in comparison with the receptor complex Z45617795. Promising drugs of MM/GBSA analysis showed several interactions in the active site of SARS-CoV-2 M^pro^. Accordingly, these drugs might be considered for a drug repurposing approach against novel coronavirus. Besides, 17 experimental drugs were found to possess a higher docking score than both the controls during virtual screening and might be vital for novel drug development against COVID-19. Furthermore, the docked complex had a strict nature in the simulated environment. However, these drugs required further investigation using in vitro and in vivo study to confirm their activity against COVID-19.

## Figures and Tables

**Table 1:** Sequence comparison matrix of SARS-CoV-2 (PDB ID: 5R7Y), SARS-CoV (PDB ID: 2C3S), and MERS-CoV (PDB ID: 5C3N). Identity matrix is expressed as a percentage.

Sequence Name	5R7Y	2C3S	5C3N
**5R7Y**	100	96	24.2
**2C3S**	96	100	25.5
**5C3N**	24.2	25.5	100

**Table 2:** Docking score of approved and investigational drugs.

Drug Bank ID	Drug Name	Group	Category	Docking Score (kcal/mol)
DB08797	Salicylamide	Approved	Analgesic	−7.10
DB13026	Ornidazole	Investigational	Antibiotic	−6.67
DB14575	Eslicarbazepine	Approved	Anticonvulsants.	−6.56
DB14855	2-(aminomethyl)phenol	Investigational	Benzene Derivatives	−6.52
DB13136	Fluindione	Approved	Anticoagulants	−6.34
DB04571	Trioxsalen	Approved	Vitiligo	−6.34
DB02262	Orotic acid	Investigational	Pyrimidines	−6.29
DB12155	Napabucasin	Investigational	Anticancer	−6.25
DB11836	Sapanisertib	Investigational	Anticancer	−6.24
DB12804	Daniquidone	Investigational	Anticancer	−6.13
DB00368	Norepinephrine bitartrate	Approved	Cardiovascular agent	−6.12
DB01424	Aminophenazone	Approved; withdrawn	Analgesic	−6.10
DB12827	Indoximod	Investigational	Immunometabolic	−6.05
DB06408	Taribavirin Hydrochloride	Investigational	Antiviral	−6.03
DB00480	Lenalidomide	Approved	Anticancer	−5.99
DB01033	Mercaptopurine monohydrate	Approved	Anticancer	−5.99
-	Z45617795	-	Control 1	−5.367
-	Inhibitor N3	-	Control 2	−4.561

**Table 3:** Docking score of experimental drugs.

Drug Bank ID	Drug Name	Docking Score (kcal/mol)
DB02502	8-hydroxy-2′-deoxyguanosine	−7.29
DB02309	5-monophosphate-9-beta-D-ribofuranosyl xanthine	−7.20
DB02690	8-hydroxy-2-methyl-3,4-dihydroquinazolin-4-one	−7.09
DB03730	3,9-Dimethyladenine	−6.97
DB04446	Benzo[B]Thiophene-2-Carboxamidine	−6.93
DB02599	2,6-Diamino-8-Propylsulfanylmethyl-3h-Quinazoline-4- One	−6.79
DB15622	Triazavirin	−6.76
DB04103	3-methylcytosine	−6.63
DB02187	Equilin	−6.61
DB04312	(2,3-difluorophenyl)methanol	−6.47
DB13549	4-dimethylaminophenol	−6.46
DB07206	6-[2-(1H-indol-6-yl)ethyl]pyridin-2-amine	−6.41
DB04448	(2,4-difluorophenyl)methanol	−6.13
DB02586	4,7-dimethyl-1,10-phenanthroline	−6.10
DB04586	2-bromophenol	−6.05
DB03763	5-methyl-2′-deoxypseudouridine	−6.00
DB04440	Purine nucleoside	−5.99

**Table 4:** Binding interactions of seven drugs obtained through MM/GBSA analysis against SARS-CoV-2 M^pro^.

Compounds	Amino Acid Residue	
	Hydrogen Bond Interactions	Hydrophobic Interactions
Sapanisertib	ASN-142	HIS-41, MET-49, MET-165
Ornidazole	ARG-188, GLN-189	HIS-41, MET-165
Napabucasin	PRO-168	MET-165
Lenalidomide	LYS-5, ARG-4	ALA-7
Daniquidone	THR-25	HIS-41, MET-49, MET-165,
Indoximod	THR-129, GlU-166	MET-165
Salicylamide	CYS-145, HIS-164, GLU-166	MET-165
Control 1	-	MET-165, MET-49, HIS-41
Control 2	SER-139, GLY138	LYS-137, LYS-5

## Supplementary Materials

The following are available online at https://www.mdpi.com/2079-7737/10/1/2/s1, Figure S1: Figure S1. The molecular dynamics simulation study of the docked complex, (A) solvent accessible surface area (SASA), and (B) radius of gyration (Rg) were assessed from the simulation trajectories.
