# Supplementary material for: Drug Repurposing Approach against Novel Coronavirus Disease (COVID-19) through Virtual Screening Targeting SARS-CoV-2 Main Protease
Source: Biology (Basel). 2020 Dec 23;10(1):2. doi: 10.3390/biology10010002 (PMC7822464; doi:10.3390/biology10010002)
Supplement: Supplementary file 1 [file biology-10-00002-s001.pdf]

biology

MDPI

Supplementary Materials

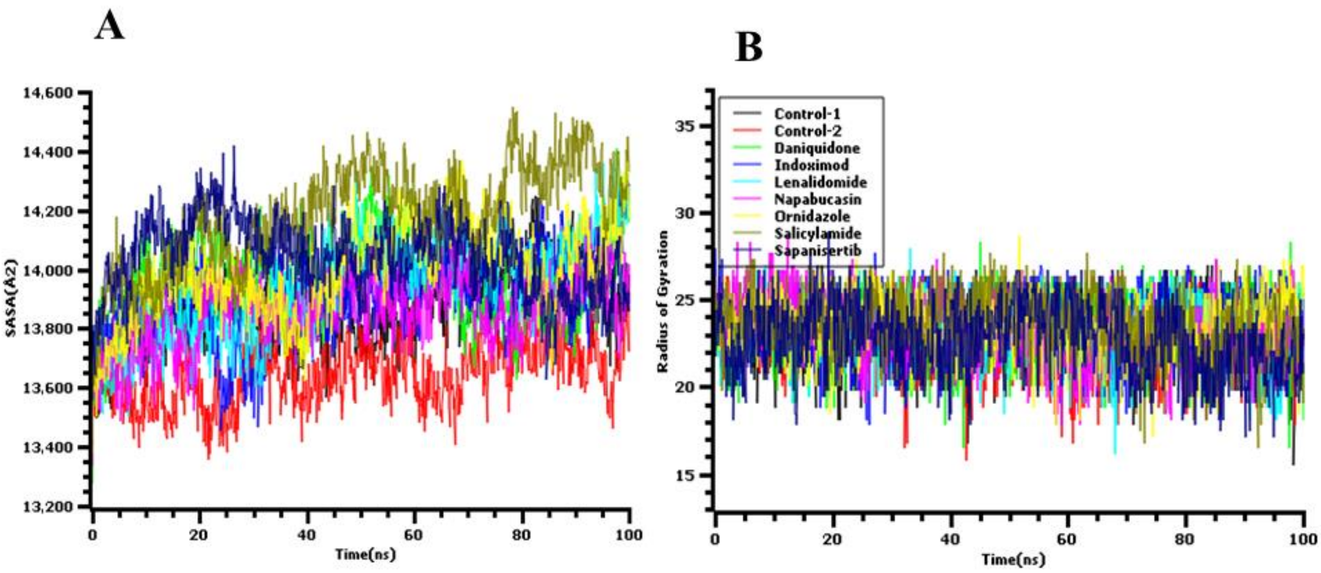

Figure S1. The molecular dynamics simulation study of the docked complex, (A) solvent accessible Scheme . urface area (SASA) and (B) radius of gyration (Rg) were assessed from the simulation trajectories.
